# Online transdiagnostic intervention for emotional, trauma- and stressor-related disorders in the Mexican population: A randomized pilot and feasibility trial

**DOI:** 10.1016/j.conctc.2023.101204

**Published:** 2023-08-25

**Authors:** Anabel de la Rosa-Gómez, Alejandrina Hernández Posadas, Pablo D. Valencia, Lorena Alejandra Flores-Plata, Berenice Serrano Zárate, Alicia Ivet Flores Elvira, Alejandro Dominguez-Rodriguez, Mario Fabián Vázquez Sánchez, Edith González Santiago

**Affiliations:** a Faculty of Higher Studies Iztacala, National Autonomous University of Mexico, Mexico; b Faculty of Health Sciences, Department of Basic and Clinical Psychology and Psychobiology, Universitat Jaume I, Spain; c Faculty of Behavioural, Management, and Social Sciences, Department of Psychology, Health and Technology, University of Twente, Enschede, the Netherlands

## Abstract

• Mexican individuals received via telehealth the unified protocol for anxiety, depression, and trauma related disorders.
• Online unified protocol reduced depression, anxiety, emotional issues, uncertainty intolerance, and psychological distress.
• The transdiagnostic unified protocol is well-suited to train therapists for emotional, trauma, and stressor-related disorders.

## 1 Background

Cognitive-behavioral interventions, from a perspective focused on specific disorders, have shown substantial empirical results and constitute the first-line treatment for anxiety and depression care [1]. However, transdiagnostic treatments have been shown to contribute to overcoming the drawbacks related to comorbidity between disorders because common or shared risk factors between disorders are addressed, including underlying mechanisms, psychological processes (cognitive, behavioral, and physiological) linked to explanatory variables, and maintenance of overlapping symptoms (attentional biases, negative thinking, avoidance, etc.), appealing to a convergent and integrative scientific approach ([[2], [3]]. propose a unified transdiagnostic behavioral cognitive protocol (Unified Protocol, UP), for the treatment of Emotional Disorders (ED) with an emphasis on emotional regulation (Ellard et al., 2010). The UP has been shown to be effective not only in achieving a reduction in anxiety and/or depression symptoms but also in increasing attendance at therapeutic sessions compared to conventional psychological interventions [4].

There are currently few studies that examine the efficacy of UP treatment for trauma and stress-related disorders, however, the evidence is promising. Findings from studies in war veterans with Posttraumatic Stress Disorder (PTSD), suggest that transdiagnostic therapy is effective on cognitive emotion regulation, avoidance, and symptom reduction compared between experimental and control groups [5]. Gutner et al. (2022) also reported improvement over time in trauma-exposed veterans across conditions (UP, present-centered therapy, or treatment as usual) with large effect sizes (range: 2.15 to −3.32), with the UP demonstrating the largest change. Regarding its suitability, clinical utility, and acceptability, the UP has shown promise for improving efficiency, satisfaction, and the personalizing of mental healthcare [6]. Meyer et al. (2022) administered the UP via videoconferencing to treat symptoms of stress-related disorders, obtaining a significant reduction in self-reported PTSD symptoms at post-treatment and large effect size improvements.

Therefore, emotion-focused transdiagnostic interventions delivered via the Internet can enhance the reach and impact of psychological treatment programs for anxiety and depression [7]. However, there are few controlled clinical studies that investigate the effects of transdiagnostic treatment via the Internet for ED adapted to the context and culture in Latin America [[8], [9]].

The general objective of this study was to determine the indicators of suitability, clinical utility, and satisfaction of a transdiagnostic online intervention for the treatment of emotional disorders and those derived from stress and trauma in a Mexican community sample. We hypothesize that: H_1_: the transdiagnostic intervention program via the internet (videoconferencing) will reduce symptoms of anxiety and/or depression and/or comorbid acute stress compared to a CBT and a WL group. H_2_: the transdiagnostic intervention program via the Internet will show statistical gains in the reduction of symptoms of anxiety and/or depression and/or acute stress, and a clinically significant improvement greater than the CBT intervention program and the WL group. H_3_: there will be a higher acceptance/satisfaction index reported by the participants in the transdiagnostic intervention program via the Internet compared to the CBT intervention and the WL group.

## 2 Method

### 2.1 Participants

The eligibility assessment consisted of the application of self-administered online questionnaires. The eligibility criteria were: a) being 18 or older; b) voluntarily participating in the study; c) meeting at least two of the following criteria: moderate overall symptomatology as measured by the Symptom Checklist (SCL-90-R; [10], mild or moderate anxiety as measured by the Beck Anxiety Inventory (BAI; Beck & Steer, 1990), mild or moderate depression as measured by the Beck Depression Inventory (BDI-II; Beck, Steer, & Brown,1996); d) having access to computer equipment with internet connection; e) having a valid email address; f) having basic digital skills in the use of an operating system and internet browsing. Participants who reported a score ≤25 (mild and moderate levels) on the BAI and/or ≤30 (mild and moderate levels) on the BDI- II were invited to an individual videoconference session to determine comorbidities with psychiatric disorders, alcohol, and drug abuse, serious medical illnesses, or suicidal behavior. The interview was carried out by supervised therapist trainees using the Mini-International Neuropsychiatric Interview [11].

As exclusion criteria the following were considered: a) current or past psychotic disorder; b) alcohol and drug abuse or dependence; c) moderate or severe risk of suicide; d) receiving psychological and/or pharmacological treatment during the study. As elimination criteria were considered: a) not accepting the conditions of informed consent and b) having missed three consecutive sessions of treatment.

### 2.2 Study design

A randomized pilot and feasibility trial was carried out with three independent groups and with repeated measures before and after the intervention: pretest and posttest. Participants were randomly assigned to three conditions: (a) transdiagnostic intervention by videoconference (UP; Barlow et al., 2011); (b) cognitive-behavioral intervention by videoconference (CBT; Flores et al., 2014) and (c) waiting list (WL). The study's trial registration number is NCT05081830.

### 2.3 Recruitment

Recruitment was carried out via advertisements in digital media (website and social networks). Participants registered through a form and were contacted by email. The interested parties were asked for their consent to participate. The coordinator asked the evaluators to blindly determine the inclusion of participants in the study based on the initial synchronous interview (via video conference) and the self-reports related to the selection criteria. Once the evaluation was completed, participants were randomly assigned to one of the three study conditions. The trial recruited participants from October 2021 to December 2022. Recruitment ceased as the target sample size was achieved.

### 2.4 Randomization and blinding

Randomization was performed by an independent investigator using the randomizer.org software in a 1:1:1 ratio. The coordinator informed the patients of their participation condition and, depending on the characteristics of the treatment condition, they were put in contact with the assigned psychotherapist. Participants in the waiting-list control group were assigned to the UP group after 2 months. All participants could withdraw from treatment at any time. The assessor, the participant and the investigator were blinded. The person who administered the initial assessments was blinded to the treatment group of the patients. This evaluator was different from the one who administered the treatment throughout the study.

### 2.5 Interventions

Experimental Condition. UP treatment was provided in eight individual sessions of 60 min, once a week via videoconference. The integrity of the treatment was controlled through the therapist's manual [12] adapted for the Mexican population and for the online modality. Each psychotherapist was assigned an expert supervisor in the intervention to monitor the adherence and application of the protocol. The main components were shown in Table 1.

**Table 1:** Sessions and activities of Transdiagnostic unified protocol (UP).

#	Session	Activities
1	Establishing goals and maintaining motivation	Analyze the benefits and costs of changing or staying the same and improving self-efficacy to increase motivation and commitment to treatment.
2	Understanding emotions	Understand the functional nature of emotions and knowledge of emotional response patterns, including the factors that maintain them.
3	Mindfulness emotional awareness	Learn to pay attention to the present moment without judging your own experiences.
4	Cognitive flexibility	Identify cognitive distortions to achieve cognitive flexibility through reinterpretation strategies.
5	Opposing emotional behaviors	Identify behaviors used to avoid unpleasant emotions and use alternative actions that come close to these emotions.
6	Understanding and coping with physical sensations	Identify physical sensations and develop tolerance to them to decrease the perceived threat.
7	Emotional exposures	Improve tolerance to unpleasant emotions through exposure.
8	Recognizing your accomplishments and looking towards the future.	Maintain the benefits of treatment in the long term and prevent relapses.

Control Condition. Non-protocol cognitive-behavioral treatment (CBT). The intervention program was brief with active participation, focused and directive, during 8 weekly individual 1-h sessions via videoconference. This program incorporates psychological techniques under the cognitive-behavioral model that have demonstrated their effectiveness in the internet modality [13,14]. The order of application of each technique was established by the psychotherapist under supervision, derived from the treatment formulation of each case to solve the problems raised. The main components are shown in Table 2.

**Table 2:** Activities of cognitive-behavioral treatment (CBT).

Components	Activities
Psychoeducation	Understanding how thoughts, emotions, and behaviors are interrelated, and how participants can learn to change unhelpful patterns.
Cognitive Restructuring	Identifying and challenging negative automatic thoughts and beliefs and replacing them with more balanced and adaptive ones.
Identification and Expression of Emotions	Recognizing and labeling emotions and learning healthy ways of expressing and managing them.
Assertiveness Training	Communicating needs, rights, and boundaries in a clear and respectful way.
Behavioral Activation	Identifying and engaging in pleasurable and meaningful activities.
Relaxation Techniques	Training in deep breathing, progressive muscle relaxation, or guided imagery, to help reduce stress and anxiety.
Problem-Solving	Identifying and solving problems in a systematic and effective way.
Relapse Prevention	Developing strategies to prevent relapse, such as identifying triggers, developing a relapse prevention plan, and practicing self-care.

No Intervention: Waiting list control (WL). Participants in the control group on the waiting list were assigned to the UP intervention after 2 months after randomization.

### 2.6 Psychotherapist training

The psychotherapists underwent UP treatment and telepsychology approach training for a total of 30 h. At the end of the training, the psychotherapist underwent a clinical competency assessment through video conference with a simulated user. During the study, the psychotherapist received individual and group supervision by professionals with experience in the field.

## 3 Measures

### 3.1 Primary outcome measures

The primary outcome measures of the trial were the self-report version of the Symptom Checklist (SCL-90-R) to screen and identify symptoms of various psychopathologies (somatization, obsessive–compulsive, interpersonal sensitivity, depression, anxiety, hostility, phobic anxiety, paranoia, and psychoticism) [10]. Also, The Posttraumatic Stress Disorder Checklist for DSM-5 (PCL-5) [15] was used to evaluate symptoms of PTSD taking into consideration the diagnostic criteria of activation, alterations, avoidance, and re-experiencing [16].

Anxiety was assessed using the Beck Anxiety Inventory (BAI) [17,18]. Ranges: minimal (0–7), mild (8–15), moderate (16–25), and severe (+26), and depression using the Beck Depression Inventory (BDI-II) [19,20] for the version II. Ranges: minimally depressed (0–13), mildly depressed (14–19), moderately depressed (20–28), and severely depressed (29–63).

Transdiagnostic Psychological processes were assessed using the Difficulties in Emotional Regulation Scale (DERS) [21,22], and Intolerance of Uncertainty Scale (IUS-12) (Carleton et al., 2007; [23].

### 3.2 *Secondary outcome measures*. *Opinion measures*

*Acceptability,* it was assessed using three questions that rated adequacy, usefulness, and applicability of the treatment to other psychological problems on a scale of 1 (“Not at all”) to 10 (“Very much”). *Suitability* was measured using two questions rated on a scale of 1 (“Not at all”) to 10 (“Very much”), which assessed how interesting the sessions were and how well the activities were understood. *Satisfaction,* it was assessed using two questions rated on a 6-point Likert scale. The first question evaluated how well the therapist treated the problem, with options ranging from “Completely dissatisfied” to “Completely satisfied”. The second question evaluated whether the treatment was helpful for the participant's specific problem, with options ranging from “Not at all” to “Made things so much better”.

### 3.3 Sample size

Was performed a sample size calculation for a one-way ANOVA with three groups using the pwr package in R. We aimed to obtain an expected power of 80% and a desired detectable effect of medium size (f = 0.25). Assuming equal sample sizes for all groups, we obtained an estimated sample size of approximately 156 participants in total or 52 participants per group. Additionally, to make focused comparisons between groups, with a medium effect size (d = 0.50) and a two-sided test, it was deemed necessary to include 64 participants per group. As a result, the final decision was to have a total sample size of 192.

### 3.4 Statistical analysis

The data analysis was conducted using R version 4.0.3 and the following R packages: rstatix (version 0.6.0), mice (version 3.13.0), miceadds (version 3.16–18), mitml (version 0.4–4), and emmeans (version 1.8.6). The aim of the analysis was to compare the effect of three treatment conditions. A complete-case analysis was conducted first, followed by multiple imputation to address missing data. The imputation model included the group variable and the pretest data as predictors to impute missing values. Two different analytical approaches were used to evaluate treatment effects: per-protocol and intention-to-treat. The per-protocol analysis was based on the subset of participants who completed the treatment as well as the posttest assessment, while the intention-to-treat analysis included all randomized participants, regardless of whether they completed the treatment or not.

Analysis of variance (ANOVA) was used to test for overall treatment effects and marginal means were used to compare differences between groups. Effect size was measured using eta-squared (η^2^). The emmeans package was used to estimate marginal means. Results of the analysis were reported separately for the per-protocol and intention-to-treat approaches. Both approaches were compared to examine the robustness of the findings.

## 4 Results

### 4.1 Characteristics of the participants

**Fig. 1:**
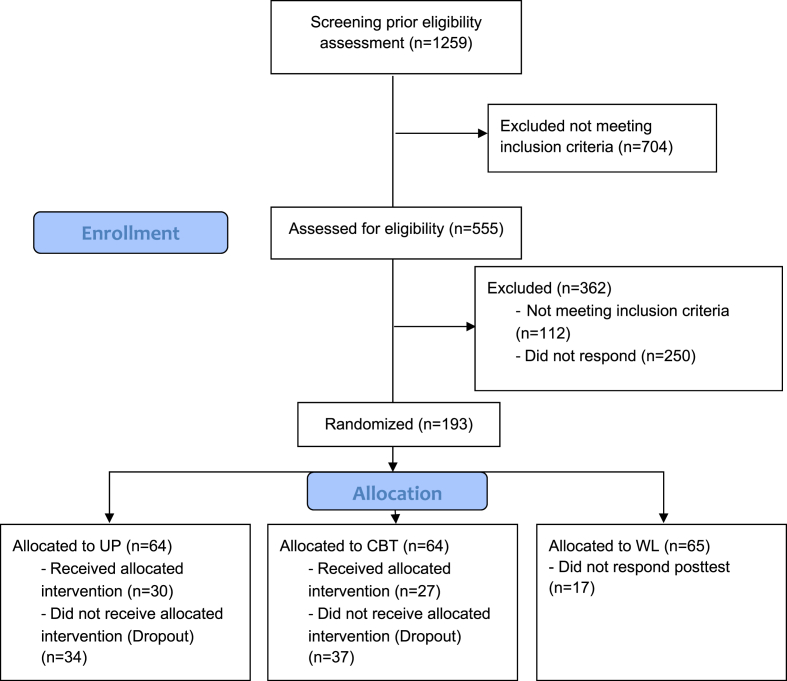
Flow Diagram of participants.

A screening process was initially conducted to determine eligibility for assessment. A total of 1259 participants were assessed through an online survey administered via SurveyMonkey and promoted via digital social media. Out of the participants, 704 were excluded from the study as they did not meet the inclusion criteria. 104 were identified as having minimal symptoms of anxiety and depression and were therefore classified as having no risk. On the other hand, 600 participants were classified at risk due to exhibiting severe symptoms in at least one measurement. Out of these, 555 individuals were chosen for further evaluation using the Mini-International Neuropsychiatric Interview, version 5.0 [11]. After the screening process, 193 individuals met the requirements to be included and were randomized (Fig. 1). Participants who voluntarily agreed to join the study had an average age of 32.65 (*SD* = 11.48) years old and were aged between 18 and 70 years, 87% women. All the participants presented anxious, depressive, and/or post-traumatic stress symptomatology and were selected by non-probabilistic, intentional, subject-type sampling and were randomly assigned to three conditions (UP, CBT, WL): a) transdiagnostic treatment under the UP via videoconference (n = 64); b) CBT, without an established protocol via videoconference (n = 64) and c) waiting list (WL) (n = 65).

### 4.2 Comparison of clinical symptoms between groups at the pretest

Baseline sociodemographic variables and outcome measures of the total sample as well as for each group separately by treatment condition are provided in Table 3. At baseline, there were no statistically significant differences in age (*F* = 0.72, *p* = .490, η^2^ = .01), sex (χ^2^ = 4.60, *p* = .100), civil status (χ^2^ = 6.20, *p* = .185) and occupation (χ^2^ = 6.09, *p* = .413). There was a slight difference in terms of educational level, as a lower proportion of people with higher education was observed in the UP condition (χ^2^ = 6.97, *p* = .031). There were no significant differences in the outcome measures, including SCL-90-R (*F* = .469, *p* = .625, η^2^ = .01), PCL-5 (*F* = .812, *p* = .446, η^2^ = .01), DERS-14 (*F* = 1.154, *p* = .318, η^2^ = .01), BAI (*F* = 1.99, *p* = .138, η^2^ = .02), BDI-II (*F* = .165, *p* = .848, η^2^ = 0). The only exception was IUS-12, which had a marginally significant difference between groups (*F* = 3.143, *p* = .045, η^2^ = .03); this, however, was of a negligible size and consistent with random allocation.

**Table 3:** Sociodemographic characteristics and baseline outcome measures of the total sample and by experimental condition.

	Total (*n* = 193)	UP (*n* = 64)	CBT (*n* = 64)	WL (*n* = 65)
Age, *M* (*SD)*	32.65 (11.48)	31.53 (11.28)	32.50 (9.46)	33.92 (13.37)
Sex, *n* (%)				
Women	168 (87)	58 (90.6)	51 (79.7)	59 (90.8)
Men	25 (13)	6 (9.4)	13 (20.3)	6 (9.2)
Civil status, *n* (%)				
Single	99 (51.3)	31 (48.4)	35 (54.7)	33 (50.8)
Married or living with a partner	69 (35.8)	28 (43.8)	22 (34.4)	19 (29.2)
Other	25 (13.0)	5 (7.8)	7 (10.9)	13 (20.0)
Higher education, *n* (%)				
No	55 (28.5)	26 (40.6)	15 (23.4)	14 (21.5)
Yes	138 (71.5)	38 (59.4)	49 (76.6)	51 (78.5)
Occupation, *n* (%)				
Student	62 (32.1)	20 (31.2)	22 (34.4)	20 (30.8.1)
Employee	38 (19.7)	16 (25.0)	13 (20.3)	9 (13.8)
Professional	28 (14.5)	5 (7.8)	11 (17.2)	12 (18.5)
Other (homemaker, unemployed, self-employed, retired, or other)	65 (33.7)	23 (35.9)	18 (28.1)	24 (36.9)
Outcomes, *M* (*SD)*				
SCL-90-R	1.78 (0.45)	1.78 (0.43)	1.82 (0.50)	1.74 (0.40)
PCL-5	39.93 (12.01)	40.61 (11.65)	40.81 (12.26)	38.38 (12.14)
DERS-14	39.50 (11.43)	39.17 (11.09)	41.17 (12.27)	38.17 (10.86)
BAI	19.45 (7.72)	20.92 (8.70)	18.25 (7.31)	19.17 (6.93)
BDI-II	26.68 (8.12)	26.27 (6.95)	27.09 (9.19)	26.69 (8.17)
IUS-12	48.66 (10.27)	46.92 (9.79)	51.20 (9.42)	47.86 (11.16)

*Note*: UP= Unified Protocol Group; CBT= Cognitive Behavioral Therapy Group; WL= Waiting list.

### 4.3 Effects of the intervention: comparisons between groups at posttest

Out of the 193 initial participants who were randomly assigned in the study, 105 (54.4%) completed the treatment as well as the posttest assessment. The complete case means and standard deviations for the pretest and post-test of the outcome measures are presented in Table 4 and the post-test comparisons and effects sizes are in Table 5, Table 6. Overall, there was a significant effect of the intervention for both treatment groups compared to the WL control group. However, there were no statistically significant differences between the treatment groups. This pattern of results was observed in all outcome measures. The between-group effect sizes were significant across all outcome measures. Effect sizes were larger for the BDI-II (η^2^ = .52), the DERS-14 (η^2^ = .46), and the SCL-90-R (η^2^ = .43), indicating a greater impact of the interventions on depression, emotional dysregulation, and psychological distress. On the other hand, the effect sizes for the BAI (η^2^ = .25) and the IUS-12 (η^2^ = .23) were small, suggesting a smaller impact on anxiety and intolerance of uncertainty. Overall, the largest effect size was for depression, which resulted in moderate to minimal average symptom reductions. Nevertheless, anxiety symptoms were also reduced; the average level changed from moderate to mild.

**Table 4:** Complete case means and standard deviations for pretest and posttest.

Outcome	UP (*n* = 30)		CBT (*n* = 27)		WL (*n* = 48)	
	Pretest	Posttest	Pretest	Posttest	Pretest	Posttest
SCL-90-R	1.75 (0.39)	0.69 (0.59)	1.81 (0.47)	0.69 (0.53)	1.75 (0.42)	1.68 (0.59)
PCL-5	38.23 (11.93)	17.17 (15.72)	39.11 (13.96)	14.00 (10.47)	38.60 (12.13)	36.81 (14.55)
DERS-14	36.60 (11.16)	21.87 (8.29)	40.30 (13.10)	21.30 (7.63)	39.42 (11.46)	39.19 (11.39)
BAI	19.97 (8.87)	10.20 (9.97)	17.67 (7.79)	8.48 (9.65)	19.21 (7.38)	20.26 (9.36)
BDI-II	25.73 (5.88)	9.40 (10.13)	25.22 (8.00)	8.33 (9.03)	26.04 (8.15)	29.21 (10.21)
IUS-12	46.67 (10.92)	37.20 (12.31)	50.89 (9.08)	37.63 (9.98)	47.75 (12.18)	49.44 (10.87)

*Note*: UP= Unified Protocol Group; CBT= Cognitive Behavioral Therapy Group; WL= Waiting list.

**Table 5:** Mean scores and one-way ANOVAs comparing experimental and wait-list control groups.

Outcome	Completers estimates						Multiple imputation estimates					
	UP (*n* = 30)	CBT (*n* = 27)	WL (*n* = 48)	*F*	*p*	η^2^	UP (*n* = 64)	CBT (*n* = 64)	WL (*n* = 65)	*F*	*p*	η^2^
SCL-90-R	0.69	0.68	1.68	38.84	<.001	.43	0.68	0.66	1.67	36.80	<.001	.42
PCL-5	17.17	14.00	36.81	29.81	<.001	.37	17.88	14.44	36.48	26.93	<.001	.35
DERS-14	21.87	21.30	39.19	42.85	<.001	.46	22.30	21.58	38.62	37.20	<.001	.43
BAI	10.20	8.48	20.26	16.70	<.001	.25	10.64	9.05	20.45	14.59	<.001	.23
BDI-II	9.40	8.33	29.21	54.38	<.001	.52	9.56	8.92	29.39	30.61	<.001	.48
IUS-12	37.20	37.71	49.44	15.51	<.001	.23	37.15	37.90	49.61	18.75	<.001	.23

**Table 6:** Marginal means’ pairwise comparisons of the clinical outcome measures.

Outcome variable	Comparison	Completers estimates			Multiple imputation estimates		
		Marginal Mean Difference [95% CI]	*p*	*d* [95% CI]	Marginal Mean Difference [95% CI]	*p*	*d* [95% CI]
SCL-90-R	Waitlist vs. UP	1.00 [0.68, 1.32]	<.001	1.73 [1.21, 2.25]	0.99 [0.71, 1.28]	<.001	1.78 [1.31, 2.24]
	Waitlist vs. CBT	1.00 [0.67, 1.33]	<.001	1.73 [1.19, 2.26]	1.01 [0.72, 1.30]	<.001	1.81 [1.34, 2.28]
	UP vs. CBT	0 [-0.36, 0.37]	1	0 [-0.53, 0.53]	0.02 [-0.28, 0.32]	.990	0.03 [-0.41, 0.48]
PCL-5	Waitlist vs. UP	19.64 [11.87, 27.40]	<.001	1.41 [0.90, 1.91]	18.60 [11.50, 25.70]	<.001	1.39 [0.93, 1.85]
	Waitlist vs. CBT	22.81 [14.78, 30.80]	<.001	1.63 [1.10, 2.16]	22.04 [15.10, 29.00]	<.001	1.65 [1.18, 2.11]
	UP vs. CBT	3.17 [-5.66, 12.00]	0.671	0.23 [-0.30, 0.75]	3.44 [-4.00, 10.90]	.520	0.26 [-0.21, 0.72]
DERS-14	Waitlist vs. UP	17.32 [11.95, 22.69]	<.001	1.79 [1.26, 2.31]	16.32 [11.44, 21.20]	<.001	1.76 [1.29, 2.34]
	Waitlist vs. CBT	17.89 [12.34, 23.44]	<.001	1.84 [1.30, 2.39]	17.03 [12.31, 21.76]	<.001	1.84 [1.38, 2.31]
	UP vs. CBT	0.57 [-5.55, 6.69]	.973	0.06 [-0.47, 0.59]	0.72 [-4.32, 5.75]	.940	0.08 [-0.38, 0.53]
BAI	Waitlist vs. UP	10.06 [4.71, 15.40]	<.001	1.05 [0.56, 1.53]	9.81 [4.68, 14.90]	<.001	1.03 [0.57, 1.49]
	Waitlist vs. CBT	11.77 [6.25, 17.30]	<.001	1.23 [0.72, 1.73]	11.39 [6.34, 16.40]	<.001	1.20 [0.74, 1.65]
	UP vs. CBT	1.72 [-4.35, 7.78]	.779	0.18 [-0.35, 0.71]	1.58 [-3.84, 7.00]	.770	0.17 [-0.31, 0.64]
BDI-II	Waitlist vs. UP	19.81 [14.31, 25.31]	<.001	2.00 [1.46, 2.54]	19.83 [14.42, 25.24]	<.001	1.98 [1.49, 2.48]
	Waitlist vs. CBT	20.88 [15.19, 26.56]	<.001	2.11 [1.55, 2.67]	20.47 [14.97, 25.97]	<.001	2.04 [1.54, 2.55]
	UP vs. CBT	1.07 [-5.18, 7.31]	.913	0.11 [-0.42, 0.63]	0.64 [-5.38, 6.66]	.966	0.06 [-0.44, 0.57]
IUS-12	Waitlist vs. UP	12.24 [6.13, 18.35]	<.001	1.11 [0.62, 1.60]	12.46 [7.01, 17.91]	<.001	1.17 [0.73, 1.61]
	Waitlist vs. CBT	11.72 [5.48, 17.96]	<.001	1.06 [0.57, 1.56]	11.72 [6.24, 17.19]	<.001	1.10 [0.66, 1.54]
	UP vs. CBT	−0.51 [-7.41, 6.38]	.983	−0.05 [-0.57, 0.48]	−0.75 [-6.71, 5.22]	.953	−0.07 [-0.54, 0.40]

*Note. d* = standardized mean difference.

To address potential bias due to high dropout rates in the study, intention-to-treat analyses were conducted including all randomized participants. Missing data were imputed using the pre-treatment score as well as the treatment group as predictor variables. Overall, the results from the intention-to-treat analysis were consistent with the per-protocol analysis (Table 5, Table 6). Changes from the pretest to the posttest were statistically significant for both UP and CBT treatment groups, as well as the WL control group. This was observed across all outcome measures. Intention-to-treat effect sizes (η^2^) were slightly smaller than those observed in the per-protocol analysis.

Regarding acceptability, satisfaction, and suitability results are presented in Table 7. While mean values are similar in both complete-case and multiple-imputation estimates, standardized mean differences (Cohen's *d*) are lower (and have wider confidence intervals) in the intention-to-treat analysis. This is possibly due to increased variance in imputed datasets. All in all, the results show no significant differences between both treatment groups.

**Table 7:** Mean scores and independent T-Tests comparing experimental groups.

Outcome	Completers estimates							Multiple imputation estimates						
	UP (*n* = 30)	CBT (*n* = 27)	Mean difference [95% CI]	*t*	*df*	*p*	*d* [95% CI]	UP (*n* = 64)	CBT (*n* = 64)	Mean Difference (95% CI)	*t*	*df*	*p*	*d* [95% CI]
Acceptability	9.16	9.60	−0.45 [-0.93, 0.03]	−1.89	54.6	.064	−0.50 [-1.04, 0.04]	9.16	9.57	−0.41 [-1.14, 0.32]	−1.17	20.7	.257	−0.48 [-1.29, 0.34]
Satisfaction	5.70	5.48	0.22 [-0.16, 0.60]	1.14	43.8	.261	0.31 [-0.23, 0.84]	5.68	5.50	0.18 [-0.39, 0.75]	0.66	20.1	.520	0.27 [-0.54, 1.07]
Suitability	9.50	9.80	−0.30 [-0.59, 0]	−2.03	53.1	.047	−0.53 [-1.07, 0.01]	9.50	9.78	−0.28 [-0.72, 0.17]	−1.30	20.0	.209	−0.53 [-1.33, 0.28]

We also conducted additional analyses that controlled for baseline scores, as well as sex and age. These adjusted results were virtually identical to the unadjusted comparisons presented here, so we decided not to include them due to space limitations. However, they are available from the corresponding author upon request.

### 4.4 Dropout information

A total of 88 participants (45.6%) dropped out of the study: 34 (53.1%) dropped out from the UP-treatment group, 37 (57.81%) dropped out from the CBT treatment group and 17 (26.2%) dropped out from the WL group. A subgroup of the participants who dropped out of the intervention answered a follow-up survey to explore the reasons for dropping out. The main reasons reported for dropping out were their perception of the therapist, unclear aspects of the therapy process, and issues related to their diagnosis.

## 5 Discussion

The main results obtained show preliminary findings to evaluate the clinical utility of the treatment based on the UP via videoconference to reduce anxious and/or depressive and/or trauma- and stressor-related symptoms in a sample of Mexican participants. Results indicated that the transdiagnostic videoconference (UP) intervention program reduced anxiety and/or depression and/or trauma- and stressor-related symptoms compared to a CBT and WL group. However, the UP treatment did not show statistically and clinically significant differences with respect to the CBT intervention program. This could be explained because both treatments are based on the cognitive-behavioral approach and the difference lies in the fact that the UP applies the same protocol to different clinical diagnoses (anxiety/depression/trauma). It was shown that a superiority was found between the intervention groups compared to the WL group. Also, a good acceptance/satisfaction index was reported by the participants in both treatment groups. These results show a possible potential for equally effective use when using UP for the treatment of comorbid cases.

It should be noted that statistically and clinically significant changes were found in all clinical indicator measures (depression, anxiety, emotional regulation, psychological distress, and intolerance to uncertainty) in the two treatment groups. The effect size confirmed the clinical utility of the intervention, as it was large in both treatment groups. Some authors (e.g. Ref. [24], have found similar results when measuring the efficacy of transdiagnostic CBT delivered via the Internet in cases with co-occurring symptoms of anxiety and depression. In the case of the variables of anxiety and intolerance to uncertainty, a small effect size was found. One possible explanation could be that the study was carried out during the COVID-19 pandemic, in the years 2021 and 2022, that although the Mexican population already had a vaccination scheme, isolation, and social distancing were still intermittent, work activities were partially resumed and there was still an atmosphere of uncertainty, living with the transition to “the new normality".

Thus, the results obtained agree with the reviewed literature (e.g. Ref. [25], by showing that the treatment through a psychological program applied via the Internet was efficacious in reducing the anxious and depressive symptoms of the participants, in both experimental conditions: UP and CBT. No differences were found between groups when evaluating the efficacy of the treatments; however, it is worth noting that in both treatment conditions, there was a statistically significant decrease in relation to the pretest.

### 5.1 Strengths and limitations

The study has strengths and clinical implications. First, to the best of our knowledge, this is the first Mexican randomized controlled clinical trial to apply a transdiagnostic online (by videoconference) intervention to treat EDs and Trauma, stress-related disorders. Second, this study will contribute in terms of the feasibility of applying the online intervention in different social contexts to achieve generalization or external validity. Also, the participants will be the direct beneficiaries of the results of the study by reducing anxiety and depression and trauma, stress-related symptoms and will be able to strengthen their coping skills in the face of stressful events. Among the limitations, it is important to consider the absence of follow-ups to find out the maintenance of the improvement achieved in the clinical indicators, or to find out if a long-term difference is achieved between the treatment groups. However, the research team continues to monitor the follow-up at three, six, and 12 months after finishing the intervention and it is estimated that they will be informed in future publications. Another limitation was the dropout rate; it was considerably influenced by the fact that several participants were withdrawn from the study because they met the elimination criteria of having missed three sessions during treatment, which coincides with what has been reported in the literature (e.g., Ref. [26]. However, the dropout rate in the present study was moderate (45%), 15% more than that estimated. Among the possible explanations, it stands out that the participants reported being students (32.1%) and another (33.7%) who presumably did not have work or school activity, reported complications in schedules to connect to video conferences due to academic demands that did not allow them to attend psychotherapy weekly sessions. This limitation is documented by authors such as [27]; who propose to carry out during the first sessions a communication that allows to clearly establish the meaning of the intervention, and the need for continuous work to achieve the expected changes; and also provide reminders or notifications to strengthen adherence to online psychological treatments.

### 5.2 Future studies

The gender variable had an interesting analysis behavior since the majority of the participants who completed the intervention in both treatment conditions were women. Future research should examine the effect of the UP intervention on adherence to treatment and its relationship with gender stereotypes. In order to reduce dropout rates, it is recommended for future studies dedicate a greater number of sessions to the initial modules of the unified protocol where motivational interviewing is addressed because it allows users to identify their needs and can ponder the benefits of the intervention.

## 6 Conclusions

The UP can be successfully applied to telehealth to treat anxiety, depression, and trauma-related disorders in Mexican people. Online delivery of the unified protocol (UP) reduces depression, emotional dysregulation, anxiety, intolerance of uncertainty, and psychological distress. Although more research is needed.

## Ethical conditions

The study was approved by the Research Ethics Committee of the Faculty of Higher Studies *Iztacala* of the National Autonomous University of Mexico (CE/FESI/082020/1363). The psychotherapist undertook to protect the confidentiality of the patient and user interaction records during the psychotherapeutic process. All participants signed an informed consent form that emphasized respect for the rights of confidentiality and privacy of personal data.

## Credit authorship contribution statement

ADLRG Conceptualization, Investigation, Methodology, Funding acquisition, Writing – original draft; AHP & PDV Data curation, Formal analysis, Writing – original draft, Writing – review & editing; LFP Project administration, Investigation, Writing – review & editing; BSZ & AFE Supervision; Writing – English review & editing; AD-R Writing – review, editing & ensuring format, and quality of the manuscript; MVS & EGS Supervision; Writing – review & editing.

## Funding information

This work was supported by the UNAM-PAPIIT project (IT300721). The funding institution had no role in the design of the study or in the collection, analysis, and interpretation of the data, and had no role in the writing of the manuscript.

## Availability of data and materials

The data that support the findings of this study are available on request from the corresponding author. The data are not publicly available due to privacy or ethical restrictions.

## Declaration of competing interest

The authors declare that they have no known competing financial interests or personal relationships that could have appeared to influence the work reported in this paper.
